# CRISPR/Cas9: an overview of recent developments and applications in cancer research

**DOI:** 10.1097/JS9.0000000000001081

**Published:** 2024-02-21

**Authors:** Nandibala Devi Shamjetsabam, Rashmi Rana, Priyanka Malik, Nirmal Kumar Ganguly

**Affiliations:** a Department of Biotechnology and Research, Sir Ganga Ram Hospital New Delhi; b Department of Veterinary Microbiology, College of Veterinary Science, Guru Angad Dev Veterinary and Animal Sciences University (GADVASU), Rampura Phul, Bathinda, Punjab, India

**Keywords:** cancer, CRISPR-Cas9, gene editing, cell therapy, homology-directed repair (HDR), nonhomologous end joining (NHEJ)

## Abstract

Clustered regularly interspaced short palindromic repeats (CRISPR)-CRISPR associated protein 9 (Cas9) has risen as a potent gene editing method with vast potential across numerous domains, including its application in cancer research and therapy. This review article provides an extensive overview of the research that has been done so far on CRISPR-Cas9 with an emphasis on how it could be utilized in the treatment of cancer. The authors go into the underlying ideas behind CRISPR-Cas9, its mechanisms of action, and its application for the study of cancer biology. Furthermore, the authors investigate the various uses of CRISPR-Cas9 in cancer research, spanning from the discovery of genes and the disease to the creation of novel therapeutic approaches. The authors additionally discuss the challenges and limitations posed by CRISPR-Cas9 technology and offer insights into the potential applications and future directions of this cutting-edge field of research. The article intends to consolidate the present understanding and stimulate more research into CRISPR-Cas9’s promise as a game-changing tool for cancer research and therapy.

## Introduction

Highlights

- Clustered regularly interspaced short palindromic repeats (CRISPR)-CRISPR associated protein 9 (Cas9) technology allows precise genes modifications.
- CRISPR-Cas9 enhances the effectiveness of cancer immunotherapies.
- Enable in identifying the roles of various genes in cancer development and progression.
- Advances the development of personalized cancer therapy.

Cancer is a global health issue affecting millions worldwide. Various treatments like surgery, radiation, chemotherapy, and combination therapies have been used to identify its underlying processes. However, alternative approaches have emerged due to postsurgery recurrence, resistance, and side effects. Cancer is a genome-related disease involving gene alterations and epigenome dysregulation^1^. Understanding the impact of genetic changes, cellular adaptations, and microenvironment changes on cancer treatment is crucial for advancement. High-throughput sequencing technology, specifically the clustered regularly interspaced short palindromic repeats (CRISPR)-CRISPR associated protein 9 (Cas9) system, has revolutionized cancer research by identifying genes linked to cancer initiation and progression, enhancing genetic alteration and functional genomics, thus transforming cancer research and improving treatment efficacy.

Genome editing technology aims to incorporate site-specific modifications into intracellular DNA sequences using DNA repair mechanisms. This involves insertions, deletions, substitutions, and integrations of genes. The field of genome editing research began in the 1970s with the discovery of restriction enzymes and genome engineering^2–6^. The first breakthrough of gene editing was demonstrated in mammalian cell in which an exogenous segment of DNA was integrated into the host genome through homologous recombination (HR)^7,8^. However, the feasibility of employing only HR for genetic modification faces several limitations such as off target integration and the incorporation of DNA segment into desired location was very low^9,10^. Researchers used alternative approaches to overcome limitations, leading to a breakthrough in introducing a double strand break (DSB) at a specific target site. Additionally, a specific restriction enzyme was introduced, cutting the double-stranded DNA sequence at specific sites, achieving both HR and nonhomologous end joining (NHEJ)^11^. Therefore, researchers are exploring gene editing techniques to create specific DSBs, using four mechanisms: Meganucleases (MegNs), Zinc finger nucleases (ZFNs), Transcription activator-like effector nucleases (TALENs), and CRISPR-Cas9. CRISPR-Cas9 is a precise, efficient, cost-effective, and user-friendly genome editing technology that offers a wider range of access for scientists compared to other methods. Additionally, CRISPR-Cas9 is a groundbreaking gene editing tool that enables simultaneous editing of multiple genes, making it ideal for studying biological systems and potentially treating genetic diseases and cancer by correcting defective genes^12^. CRISPR-Cas9 technology has revolutionized cancer research and therapy by allowing precise genome changes with remarkable accuracy. This technology offers immense potential for new treatments, improved patient outcomes, and transforming our understanding of cancer^13,14^. In this review, we offer a thorough summary of the CRISPR-Cas9 research that has been conducted so far with a focus on how it could be used to treat cancer. We go into CRISPR-Cas9’s conceptual foundations, its methods of action, and its use in the study of cancer biology.

## Clustered regularly interspaced short palindromic repeats (CRISPR)-CRISPR associated protein 9 (Cas9)

CRISPR-Cas9 were first discovered in the DNA sequence of *Escherichia coli* bacteria in 1987 by Japanese scientists Ishino *et al*.^3^. The discovery of CRISPR loci’s biological function was made in 1995 when Spanish molecular biologist Francisco Mojica discovered a similar structure in the archeal genome of *Haloferax mediterranei*, despite the difficulty in sequencing DNA fragments^15^. Mojica was one of the first to hypothesize that the found DNA repeats might be part of the bacteria and archaea immune system^16^. In 2002, the current name CRISPR was first coined by Jansen *et al*.^17^ along with the suggestion by Mojica and presence of gene associated with CRISPR repeats (cas 1–4, CRISPR- associated genes) was first described. The presence of viral DNA fragments (‘spacer’ 17–84 bases) that is separated by short palindromic repeats (23–50 bases^18^) derived from bacteriophages was discovered in 2005^19,20^. In 2007, scientists confirmed the CRISPR system’s mechanism by tracing the acquisition of spacers at CRISPR loci, which mediate adaptive immunity to bacteriophages^21^. In 2008, a team led by John van der Oost at Wageningen University reported the existence of crRNA (CRISPR-associated RNA) molecules, which are the first products of transcription from the CRISPR locus and contain several spacers and repetitions^22^. In 2011, Emmanuelle Charpentier’s team discovered tracrRNA (trans-activating CRISPR RNA), a crucial RNA molecule for nuclease action, involved in crRNA processing^23^. The discovery of tracrRNA’s involvement in cleaving target DNA earned the Nobel Prize, and the notion of merging two RNA molecules into a single chimeric molecule (sgRNA) was presented, considerably improving the practical application of the CRISPR-Cas9 system in *in-vitro* research^24^. In 2013, Cong and Mali, among others, verified the utilization of the type II Cas system for the precise modification of DNA in mammalian cells^25,26^, which opened the door for using the CRISPR-Cas9 system to modify genomes. In that same year, the mutant Cas9 protein dCas9 (Cas9 with a deficient endonuclease function), was innovated, having lost its nuclease activity^27^. The dCas9 protein was fused with transcription modulators to create CRISPR activation (CRISPRa) and inhibition (CRISPRi) tools for gene transcription stimulation or suppression^28,29^. CRISPR-Cas9 and APOBEC (cytosine deaminase) were combined to modified Cas9 that makes the C-T (or G-A) conversion while being guided by gRNA without resulting in DSB by Komor *et al*.^30^. The base editor, Adenine Bases Editor (ABE), is designed to rectify point mutations in the genome, addressing disruptions in CRISPR-Cas9 and converting A-T base pairs into G-C base pairs^31^. Gilpatrick and colleagues developed third-generation nanopore sequencing using Cas9 and adapter ligation to create a nanopore Cas9-targeted sequence (nCATS), enabling cost-effective reading of large genomic segments by altering the target genome structure and specific DNA region^32^. In 2020, the ‘vfCRISPR’ CRISPR technique revolutionized DNA research by creating double-stranded breaks at small scales. This caged RNA technique allows Cas9 to attach to DNA without breaking it before light-induced activation. This precise technique can alter one allele at a time, providing valuable insights for examining genetic characteristics and cancer treatments^33^.

## Mechanisms of CRISPR-Cas9 system

**Figure 1:**
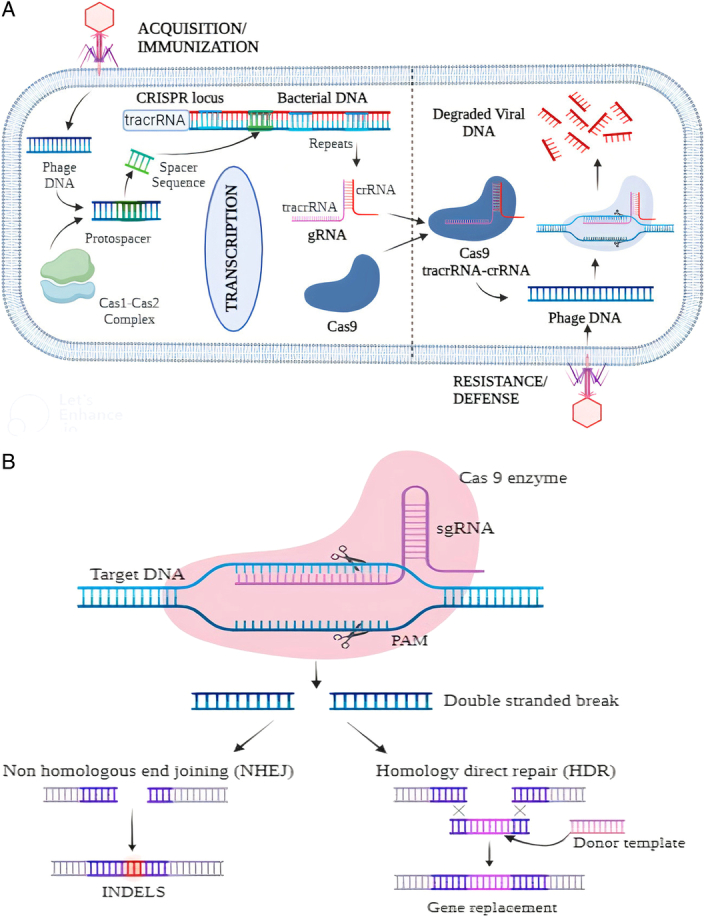
A, Biology of Natural CRISPR/Cas9 system. B, Mechanism of CRISPR/Cas9 gene editing system and double-stranded break repaired by Nonhomologous end joining (NHEJ) or Homology direct repair (HDR).

The CRISPR-Cas9 system originates from the natural adaptive immune system present in bacteria and archaea^21^. Bacteria employ CRISPR-Cas9 as a defense mechanism against viruses and other foreign genetic components^33^. It consists of a guide RNA (gRNA) and a Cas9 protein, which are designed to match the target DNA sequence. Natural immunity to this system involves three stages: acquisition, expression, and resistance, ensuring effective DNA cleavage at the designated site^34,35^ (Fig. 1). CRISPR systems create a specific immunity mechanism by incorporating protospacers (DNA fragments) from invading bacteria, into the CRISPR locus. These protospacers are transcribed and processed to produce CRISPR RNAs (crRNAs), which combine with a trans-activating CRISPR RNA (tracrRNA) and an effector protein. The crRNA forms base pairs with both DNA and tracrRNA. In some systems, tracrRNA is not involved, and foreign DNA is loaded during the resistance stage. CRISPR locus is encoded by effector protein subunits. RNase III action leads to the creation of the mature crRNA:tracrRNA complex, which is then followed by Cas9 binding, R loop formation, and endonuclease activity. The HNH and RuvC domains of the *Streptococcus pyogenes* CRISPR-Cas9 (SpCas9) protein can cleave both complementary and noncomplementary strands^36–38^.

The CRISPR-Cas9 mechanism operates in three stages: target recognition, DNA cleavage, and repair. The first stage involves the guide RNA (gRNA) attaching to the target DNA sequence, directing the Cas9 protein to its precise destination. The gRNA and SpCas9 protein form a complex scanning DNA for the protospacer adjacent motif (PAM). The Cas9 protein cleaves DNA at a specific point after finding the PAM, causing a double-stranded break. This results in the loss of the targeted gene’s function. The cell then attempts to repair the injury through either NHEJ or homology-directed repair (HDR) mechanisms. NHEJ is a quick, error-prone repair method that introduces small insertions or deletions (indels) into the DNA sequence, leading to gene disruption, while HDR is a more precise repair method that uses a template DNA molecule to repair the break^24,25,39–41^.

## CRISPR-Cas9 in cancer research

**Figure 2:**
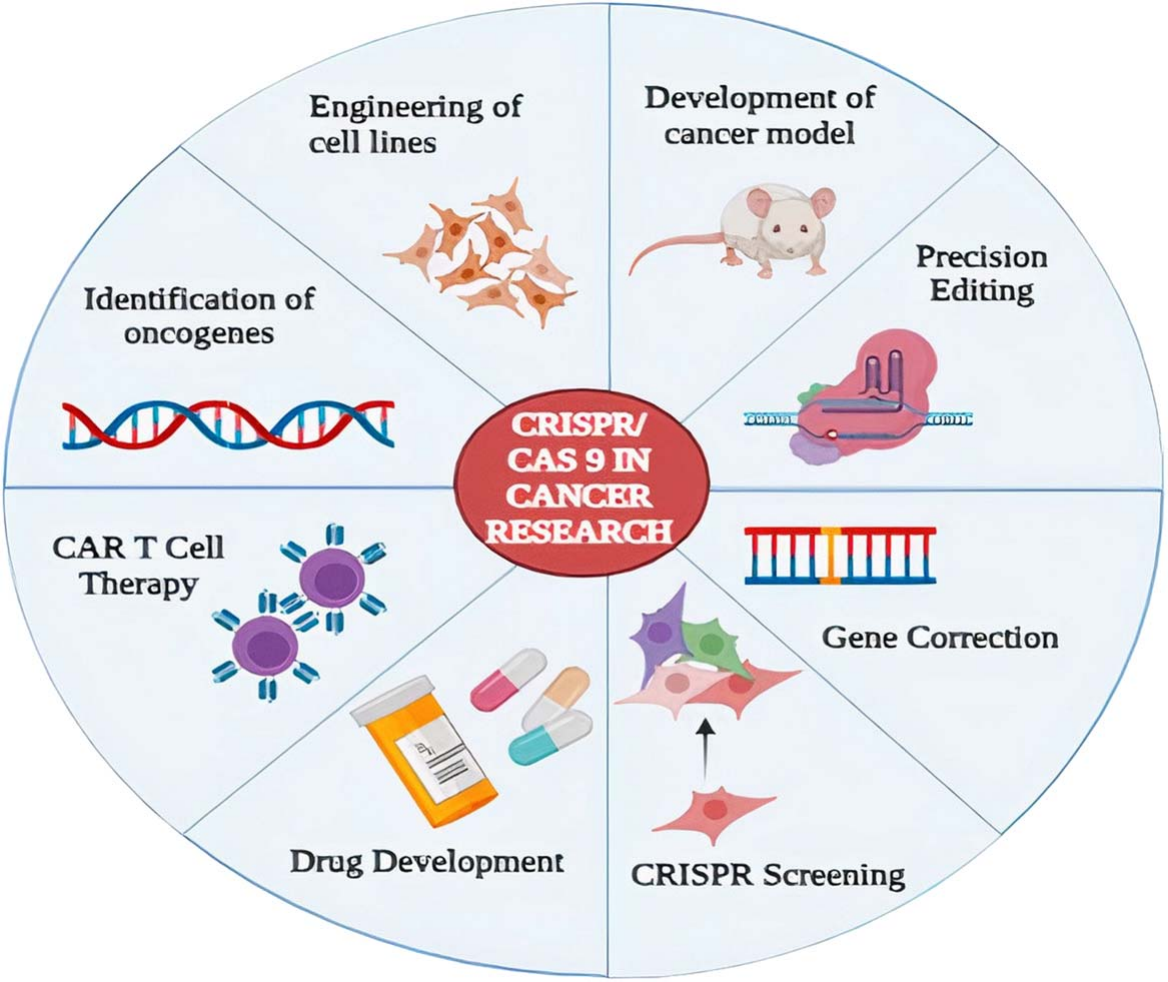
Applications of CRISPR/Cas9 in cancer research.

Cancer is a global health issue with diverse diseases. Understanding the precise genes and molecular pathways driving tumorigenesis is crucial for developing effective therapies. CRISPR-Cas9 technology has revolutionized genetic screening, allowing researchers to identify oncogenes and tumor suppressor genes with precision and efficiency (Fig. 2).

### Identification of cancer associated genes

Researchers use CRISPR-Cas9 libraries for gain-of-function screening to identify oncogenes. These libraries contain thousands of gRNAs and are inserted into cancer cell lines or *in-vivo* systems to target specific genes. Selection pressures are applied to identify genes that offer growth advantages or drug resistance. Statistically significant enrichment of gRNAs helps identify genes supporting oncogenic behavior^42^. The effectiveness of CRISPR-based screens to find oncogenes was established in a landmark work by Hart *et al*.^43^. Shalem *et al*.^44^ used the Genome-wide CRISPR-Cas9 knockout library (GecKO) that was constructed with sgRNA library targeting 18 080 human genome genes, to screen candidate genes for response to vemurafenib in melanoma cell line A375 and found six genes- TADA1, TADA2B, NF1, NF2, MED12, and Cul3. Chen *et al*.^45^ conducted a genome-wide CRISPR-Cas9 loss-of-function screen named mGeCKOa that contains 27 405 sgRNAs that target 1175 microRNA precursors and 20 611 protein-coding genes in a mouse model of tumor evolution and identified five metastasis-suppressive genes and two microRNAs. Behan *et al*. utilized CRISPR-Cas9 knockout libraries to identify new tumor suppressor genes in cancer cell lines. They identified established suppressors like TP53 and new possibilities like CIC, which were not previously associated with cancer, by carefully knocking off each gene and evaluating cell viability^46^. This study demonstrates the ability of CRISPR-based screens to find previously unknown tumor suppressor genes, thereby expanding our knowledge of cancer biology.

CRISPR-Cas technology has made it simpler to explore cancer vulnerabilities, referring to specific genetic or functional dependencies that cancer cells heavily depend on for survival. Scientists are using CRISPR-Cas9 to identify weaknesses in genes that can be exploited for therapeutic purposes. One such weakness is synthetic lethality, where two genes are disrupted simultaneously, killing cancer cells while sparing healthy ones. This led to the development of PARP inhibitors like olaparib, which have shown exceptional effectiveness in treating BRCA-mutated ovarian and breast cancers^47,48^. Here are some of the oncogenes identified through CRISPR/Cas9 and the genes that hold promise for targeted modification (Table 1).

**Table 1:** The oncogenes identified through CRISPR/Cas9 and the genes that hold promise for targeted modification.

Oncogenes	Methods	Functions	Cancer types	References
CD133	Knockout	Downregulated vimentin expression, cell proliferation, and colony formation, and inhibited cell migration and invasion	Colon cancer cells	^49^
E3 ubiquitin ligase UBR5	Deletion	Inhibited tumor growth and metastasis	Triple-negative breast cancers	^50^
miR-3064	Knockdown	Inhibited cell proliferation, invasion, and tumorigenic capacity	Pancreatic cancer cells	^51^
Mutant EGFR	Knockdown	Inhibited growth and proliferation	Lung cancer cell lines	^52^
FAK gene with KRAS mutation	Knockdown	DNA damage and increased sensitivity to radiotherapy	NSCLC cells	^53^
Tumor suppressor genes	Methods	Functions	Cancer types	References
NF2	Knockout	Enhanced migration and invasion abilities	Mesothelial cell lines	^54^
MFN2	Knockout	Enhancement of migration and invasion abilities	Lung cancer cells	^55^
P53, Nf1, Pten, Ptch1	Deletion	Preventing tumor formation	Glioblastoma (Mouse brain)	^14^
LATS1/2	Deletion	Suppressed tumor growth	Pancreatic, prostate, breast, colon, glioma, and bladder cancers (mouse cancer cell lines)	^56^

### Gene correction and targeted gene therapy

Researchers are using CRISPR-Cas9 to precisely fix genetic abnormalities in cancer cells, potentially stopping tumor growth and restoring normal cellular function, which is often caused by uncontrolled development in tumors. In 2017, Li *et al*. published a study that demonstrated the effectiveness of CRISPR-Cas9 in reversing a cancer-related mutation, specifically the TP53 gene, which is often altered in various malignancies, including lung cancer. They successfully restored the wild-type TP53 sequence in lung cancer cells, resulting in reduced tumor development and increased cell death^57^. In addition to gene correction, CRISPR-Cas9 has shown remarkable potential in targeted gene therapy for cancer. In 2018, a study by Liu *et al*. demonstrated the potential of CRISPR-Cas9 in targeted cancer therapy. The researchers altered the PD-1 gene, which suppresses the immune response to cancer cells, boosting tumor immune evasion. By disrupting the PD-1 gene, they observed enhanced antitumour immune responses and reduced tumor growth in mouse models^58^.

Immunotherapy is another innovative approach for cancer treatment that uses the body’s immune system to selectively target and eradicate cancerous cells. However, its efficacy remains limited due to factors such as tumor diversity, tumor evasion mechanisms, and unintended side effects. Immunotherapies like immune checkpoint inhibitors and CAR-T cell therapy have shown promise in some cases. CRISPR-Cas9 provides several approaches to address these challenges and improve the effectiveness of immunotherapies. Here are some of the strategies of CRISPR-Cas9 to enhance the efficacy of immunotherapy.

#### Targeting tumor mutations

The prevalence of tumor mutations can affect how well immunotherapies work. Immunotherapies can be more precise and successful by using CRISPR-Cas9 to help identify and target certain tumor mutations. In order to develop specialised immunotherapies, researchers have employed CRISPR-Cas9 to produce personalized cancer models with particular tumor mutations.

#### Enhancing T cell function

T cells are vital in immunotherapies, and their dysfunction can lead to treatment failure. CRISPR-Cas9 can enhance T cell function by editing genes involved in activation, proliferation, and effector function. For instance, researchers have successfully used CRISPR-Cas9 to disrupt the PD-1 gene, a major immune checkpoint, in T cells, resulting in increased T cell activity and improved antitumour responses^59^.

#### Improving CAR-T cell therapy

**Figure 3:**
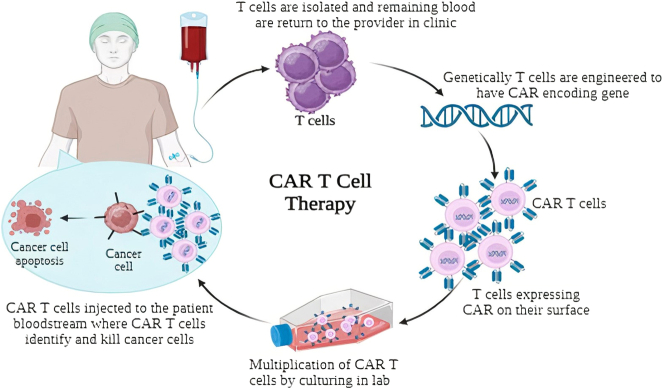
Mechanism of CAR-T Cell Therapy.

Chimeric Antigen Receptor (CAR)-T cell therapy is an innovative immunotherapy method that involves genetically altering a patient’s T cells to introduce chimeric antigen receptors (CARs), enabling them to recognize and eliminate cancer cells, demonstrating significant success in treating certain blood cancers (Fig. 3). CRISPR-Cas9 can enhance CAR-T cell therapy by improving persistence, overcoming immunosuppressive tumor microenvironments, and reducing off-target effects, but its efficacy against solid tumors remains limited^60^. Researchers have used CRISPR-Cas9 to delete genes encoding immunosuppressive molecules, such as programmed cell death protein 1 (PD-L1) or cytotoxic T-lymphocyte-associated protein 4 (CTLA-4), in CAR-T cells, enhancing tumor clearance. This allows CAR-T cells to avoid immune checkpoint inhibition, enabling sustained antitumour activity. Preclinical studies show PD-1 knockout CAR-T cells improve persistence and tumor clearance in mouse models^60^. CRISPR-Cas9 can improve CAR-T cell efficacy by modifying the CAR construct, enhancing cell expansion, persistence, and antitumour activity by introducing specific cytokines or co-stimulatory molecules^61,62^.

Clinical trials show high response rates for CAR-T cell therapy, but its effectiveness and long-term outcomes are still under investigation, with potential side effects. Cytokines release syndrome (CRS) is a condition resulting from excessive cytokine release during T-cell activation, causing flu-like symptoms, fever, hypotension, and multiorgan dysfunction. Neurotoxicity, including confusion, seizures, and cerebral edema, is another concern. However, management advancements have reduced their impact^63^. Researchers have developed strategies to minimize off-target effects in CAR-T cell therapy using CRISPR-Cas9 technology, which may edit unintended genomic loci. This is due to the potential for off-target effects, which could potentially compromise the safety profile of the therapy^64^. Additionally, the development of base editors and prime editors enables precise nucleotide substitutions without generating double-stranded breaks, minimizing the risk of off-target effects^65^. Moreover, CRISPR-Cas9 can mitigate the risk of graft-versus-host disease (GVHD) in allogeneic CAR-T cell therapy. By deleting the T-cell receptor (TCR) or human leukocyte antigen (HLA) genes, CRISPR-Cas9 can generate universal donor CAR-T cells that do not recognize host tissues, reducing the risk of GVHD^66^. This approach offers a potential solution to overcome the limitations associated with CAR-T cell therapy using patient-derived cells.

## CRISPR-Cas9 system delivery methods

**Figure 4:**
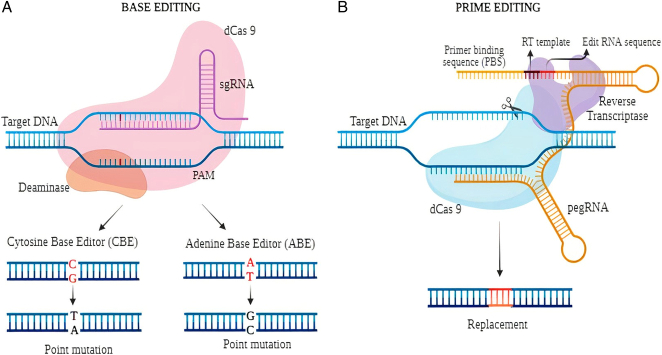
Various methods used for the delivery of the CRISPR/Cas9 system to edit the target DNA.

The CRISPR-Cas9 system is a powerful tool for genome editing, but delivering its components into target cells remains a significant challenge. This discussion explores various strategies, their benefits, constraints, and recent advancements (Fig. 4).

### Viral delivery systems

CRISPR-Cas9 is often delivered using viral vectors like lentiviruses and adeno-associated viruses (AAVs), which are effective in transporting components into various cell types. Lentiviruses have a large packaging capacity (approximately 10 kb), allowing for the inclusion of multiple Cas enzymes or guide RNAs (gRNAs) in a single vector^67^. As viral vectors for CRISPR-Cas9 delivery, lentiviruses, notably those based on the HIV, have gained attention. Lentiviral delivery faces a significant risk of insertional mutagenesis due to its integration into the host genome, requiring careful evaluation and minimized risk to ensure gene editing safety. AAVs, on the other hand, are less immunogenic and have a safer profile, making them attractive for *in vivo* applications. AAVs are harmless viruses that introduce genetic material into cells, offering high transduction efficiency, long-term transgene expression, and low immunogenicity, making them a promising delivery system^68^. Furthermore, AAVs can be engineered to target specific tissues or cell types, making them versatile tools for gene therapy applications. Recently, Herpes simplex viruses (HSVs) have emerged as an alternative viral delivery system for CRISPR-Cas9, particularly targeting the central nervous system (CNS). HSVs have a large DNA genome, allowing for the incorporation of CRISPR-Cas9 machinery, and naturally prefer neurons, making them efficient in delivering components to CNS target cells^69^. However, before clinical translation, it’s crucial to consider viral toxicity and immunogenicity risks. AAVs, lentiviruses, and HSVs have unique benefits and drawbacks, making them suitable for specific applications. Addressing viral toxicity, immunogenicity, and insertional mutagenesis is crucial for the secure and successful clinical translation of CRISPR-Cas9 based therapeutics as research progresses.

### Nonviral delivery systems

Nonviral methods, such as nanoparticles or electroporation or microinjection, are being explored as a potential replacement for CRISPR-Cas9 delivery. These methods offer benefits like decreased immunogenicity and insertional mutagenesis risk, but often have less effective delivery than viral vectors^70^. Here, we discussed the various nonviral delivery strategies, along with their advantages and limitations.

### Nanoparticle delivery methods

The method involves using nanoparticles to deliver CRISPR-Cas9 components into target cells. These nanoparticles, typically made of lipids, polymers, or inorganic materials, encapsulate the components, protecting them from degradation and facilitating efficient delivery. They can be designed to optimize cellular uptake, protect the genetic material, and facilitate endosomal escape, resulting in higher transfection efficiency. Unlike viral vectors, nanoparticles have lower immunogenicity and cytotoxicity, making them safer for therapeutic applications (Table 2).

**Table 2:** Various nanoparticle delivery systems with advantages and disadvantages.

Nanoparticle Delivery System	Advantages	Disadvantages	References
Lipid	Efficient delivery, stability, versatile delivery	Cytotoxicity, off-target effects, scalability	^71^
Polymer	Enhanced stability, improved cellular intake, and controlled release capabilities.	Cytotoxicity, off-target effects	^72^
Gold	Delivered large payloads, protecting CRISPR-Cas 9 components from degradation.	Toxicity, immunogenicity, off-target effect.	^73^
Magnetic	Size and surface properties can be precisely engineered, surface can be modified with various functional groups.	Cytotoxicity, stability, immunogenicity and long-term effect.	^74^

#### Lipid nanoparticles (LNPs) or liposomes

LNPs are used to encapsulate and deliver CRISPR components into target cells. These systems consist of a lipid bilayer mimicking the cell membrane and an aqueous core. The lipids used can be cationic, anionic, neutral, or hybrid, each with unique advantages. LNPs can be modified to enhance target specificity and reduce off-target effects. However, their efficacy depends on cell type and may suffer from poor stability and low transfection efficiency. Successful delivery of CRISPR-Cas9 components has been demonstrated using LNPs^71^. Additionally, surface modifications of LNPs, such as PEGylation, can improve their stability, circulation time, and targeting capabilities^75^. In recent years, LNPs can delivered mRNA or siRNA in preclinical trials thereby showing promising delivery method^70,76^.

#### Polymer nanoparticles

Polymer nanoparticles are a promising delivery method for CRISPR-Cas9 components, offering stability, improved cellular uptake, and protection from degradation. These nanoparticles are made from biodegradable polymers like poly (lactic-co-glycolic acid) (PLGA) or polyethyleneimine (PEI), polyethylene glycol (PEG), or chitosan which efficiently encapsulate nucleic acids. Their tunability allows for modification of size, surface charge, and drug release kinetics, enhancing their delivery efficiency^76^. Size significantly influences cellular uptake and biodistribution, with smaller particles enhancing penetration into tissues and endocytosis. Surface charge can be modulated to interact with cell membranes, facilitating cellular uptake. Surface functionalization allows for specific targeting of cell types, increasing efficiency, and specificity of CRISPR-Cas9 delivery. PLGA-based nanoparticles have successfully edited Cas9 protein and sgRNA *in-vitro* and *in-vivo* ^72^. Polymer nanoparticles have been found to be effective carriers for delivering CRISPR-Cas9. A study developed a PLGA-based nanoparticle system that encapsulated Cas9 mRNA and guide RNA, resulting in high cellular uptake, efficient endosomal escape, and successful gene editing in primary cells^77^. In a different investigation, PEGylated polymeric nanoparticles were used as carriers to deliver CRISPR-Cas9 for Duchenne muscular dystrophy (DMD), effectively reinstating dystrophin expression and enhancing muscle function in a mouse model of DMD^78^.

#### Inorganic nanoparticles

Inorganic nanoparticles, such as gold nanoparticles (AuNPs) and magnetic nanoparticles (MNPs), have gained attention as potential carriers for the CRISPR-Cas9 system. AuNPs possess unique optical properties and excellent biocompatibility, making them suitable for both delivery and imaging applications. The surface of AuNPs can be modified with DNA or RNA oligonucleotides complementary to the target DNA sequence. AuNPs are a promising delivery platform for CRISPR-Cas9 due to their ability to protect components from degradation by nucleases, ensuring their stability and functionality. Their efficient cellular uptake allows for the delivery of larger payloads, such as multiple guide RNAs or therapeutic agents, opening up new possibilities for combinatorial therapies and increasing efficacy. Studies have demonstrated successful delivery of CRISPR-Cas9 components, leading to efficient gene editing in various cell types^73^. AuNPs have potential applications in *in-vivo* CRISPR-Cas9 delivery, particularly in treating genetic disorders and cancer treatment. By editing disease-causing mutations, AuNPs offer a targeted therapeutic approach with minimal off-target effects. Additionally, AuNPs can disrupt specific oncogenes, inhibiting tumor growth and progression^79–82^.

The synthesis of MNPs involves the preparation of nanoscale metallic or metal oxide particles with dimensions ranging from 1 to 100 nm. Methods like co-precipitation, thermal decomposition, and sol-gel techniques are used to control size, shape, and magnetic properties, with the choice based on desired traits like magnetization, stability, and biocompatibility^74^. MNPs need to be functionalized to attach gene-editing components to CRISPR-Cas9, typically by conjugating biomolecules like peptides, antibodies, or nucleic acids onto their surface. This enhances stability, improves cellular uptake, and enables specific targeting of desired cell types. The functionalization can also be customized to protect CRISPR-Cas9 components during delivery, increasing efficiency and reducing off-target effects. MNPs can be utilized for targeted delivery, leveraging external magnetic fields to guide nanoparticles to specific tissues or cells^83^. Researchers have developed a method to selectively deliver CRISPR-Cas9 components to specific cell populations using MNPs functionalized with specific ligands, thereby reducing the risk of unintended gene modifications in non-target cells. Furthermore, MNPs have the potential to revolutionize gene editing by enabling real-time monitoring of delivery processes in noninvasive imaging techniques like MRI. By visualizing the precise localization of MNPs within target tissues, researchers can optimize delivery strategies and maximize CRISPR-Cas9 gene editing efficacy. MNPs have shown promise in treating genetic conditions like Duchenne muscular dystrophy and sickle cell disease, with promising early-stage experiments paving the way for clinical investigations^84^.

#### Electroporation

Electroporation is a method for introducing CRISPR-Cas9 components into cells by creating temporary pores in the cell membrane using electrical pulses. It is useful for hard-to-transfect cells like primary and stem cells due to its simplicity, versatility, and high efficiency. However, it can cause cell damage and may not be suitable for sensitive cell types^85^. Electroporation is a versatile technique for CRISPR-Cas9 delivery, allowing for efficient delivery of large-sized components like Cas9 nuclease and long gRNA sequences, making it ideal for large therapeutic genes or complex gene editing scenarios. It can be used with various cell types, enhancing the adaptability of the CRISPR-Cas9 system. Electroporation also offers high delivery efficiency, resulting in a higher proportion of successfully edited cells^86^. Emerging technology faces challenges, including optimizing electroporation parameters like voltage, pulse length, and cell density for maximum efficiency and minimizing cell damage. Thorough investigation of potential cytotoxic effects is necessary for safe clinical application in clinical settings^24^.

## FDA-Approved and clinical trials underway of CRISPR-Based cell therapy treatment for cancer

The Food and Drug Administration (FDA) approved CRISPR-based cell therapy, Kymriah, uses patients’ immune systems to combat cancer cells. This groundbreaking development, also known as tisagenlecleucel, targets CD19-positive B-cell precursor acute lymphoblastic leukemia (ALL) in children and young adults up to 25 years old who have not responded to traditional therapies. This marks a significant turning point in cancer treatment^87,88^. Novartis’ Kymriah is a chimeric antigen receptor T-cell (CAR-T) treatment that uses the patient’s immune system to fight cancer. It involves extracting and genetically modifying the patient’s T-cells to produce a specific CAR with a transmembrane domain, intracellular signaling domains, and an extracellular domain^89^. The safety and effectiveness of Kymriah were assessed in a clinical trial called ELIANA, which included 63 pediatric and young adult patients with recurrent or resistant B-cell acute lymphoblastic leukemia. The trial found that 52% of patients experienced full remission or complete remission with partial hematologic recovery 3 months after Kymriah infusion^88^. In 2018, the FDA approved Kymriah for adult patients with relapsed or refractory diffuse large B cell lymphoma (DLBCL), based on clinical trials showing significant response rates and improved overall survival. The JULIET trial, a Phase 2 single-arm trial, enrolled 93 adult patients with DLBCL, reporting an overall response rate of 50% and a complete response rate of 32%^63^. Notably, 64% of patients who achieved a complete response maintained it at a median follow-up of 14.9 months.

In August 2021, the FDA approves Abecma (idecabtagene vicleucel), an initial CAR-T cell therapy for multiple myeloma, using CRISPR/Cas9 system to alter T-cells to target and identify myeloma cells. Abecma has a 72% response rate in clinical studies, with over 50% of patients experiencing full remission. It uses a complex immune system approach to combat cancer cells, activating T-cells through the extracellular domain of the CAR, which recognizes the B-cell maturation antigen (BCMA) on myeloma cell surfaces. After infusion, modified T-cells rapidly proliferate and attack malignant cells expressing BCMA. CAR-T-cells recognize BCMA, killing cancer cells through cytotoxicity, inflammatory cytokines, and immune cell recruitment, enhancing the antitumour response and preventing further cancer growth. The Phase 2 KarMMa clinical study has shown that Abecma, a focused method for multiple myeloma patients who have tried multiple treatments, is effective. The trial included 140 individuals with relapsed or resistant multiple myeloma who had undergone at least three previous treatments. An astounding 72% of patients responded overall, with 28% attaining a strict complete response (sCR). Patient freedom from disease progression as measured by the median progression-free survival (PFS) was 8.8 months. Notably, significant results were attained in individuals who had received a lot of prior therapy and had few other choices. The KarMMa study found that CNS toxicities and CRS were the most common side events, with fevers, flu-like symptoms, and organ malfunction being hallmarks. However, with supportive treatment and the use of the interleukin-6 receptor antagonist tocilizumab, CRS incidence and severity were successfully controlled. Neurologic toxicities like encephalopathy and seizures were often treatable and manageable^90^.

The University of Pennsylvania are conducting clinical trials to evaluate the safety and efficacy of CRISPR-based cell therapy in treating B-cell cancers that have relapsed or developed resistance to standard treatments. The trials are currently in the recruitment phase and are eagerly waited for results, including overall response rate, adverse events, response duration, and PFS. Another trial conducted by scientists from the University of Texas MD Anderson Cancer Center are conducting a trial to evaluate the safety and viability of using autologous T cells modified with CRISPR-Cas9. The trial aims to assess the number and severity of adverse treatment-related events, response rate, PFS, and overall survival, with preliminary results showing promising results. The ongoing clinical trials that employ CRISPR-Cas9 for the treatment of diverse cancer types (Table 3).

**Table 3:** Ongoing clinical trials using CRISPR/Cas 9 for treatment of various cancers. (Adopted from^91^).

Clinical trial number	Phase	Target gene	Type of cancer	Type of cell
NCT03747965	1	PDCD1-Knock Out	Solid Tumor (Adult)	Mesothelin directed CAR-T cells
NCT03044743	1/2	PDCD1-Knock Out	Gastric Carcinoma (Stage IV), Nasopharyngeal Carcinoma (Stage IV), T-Cell Lymphoma (Stage IV), Adult Hodgkin Lymphoma (Stage IV), Diffuse Large B-Cell Lymphoma (Stage IV)	EBV-CTL cells
NCT03081715	1	PDCD1-Knock Out	Esophageal Cancer	Primary T-cells
NCT02793856	1	PDCD1-Knock Out	Metastatic Non-small Cell Lung Cancer	Primary T-cells
NCT04417764	1	PDCD1-Knock Out	Advanced Hepato cellular Carcinoma	Primary T-cells
NCT04426669	1/2	CISH-Knock Out	Gastrointestinal Epithelial Cancer, Gastrointestinal Neoplasms Tract Cancer, Gastrointestinal Cancer, Colorectal Cancer, Pancreatic Cancer, Gall Bladder Cancer, Colon Cancer, Esophageal Cancer, Stomach Cancer	TILs
NCT03057912	1	CISH-Knock Out	Human Papilloma virus Related Malignant Neoplasm	TILs
NCT03399448	1	TRAC, TRBC, PDCD1 Knock Out	Multiple Myeloma Melanoma Synovial Sarcoma Myxoid/Round Cell Liposarcoma	NY-ESO-1 redirected autologous T cells
NCT03545815	1	TRAC, TRBC, PDCD1 Knock Out	Solid Tumor	Anti-mesothelin CAR-T cells
NCT03166878	1/2	TRAC, TRBC, B2M-Knock Out	B-Cell Leukemia, B-Cell Lymphoma	UCART019
NCT05037669	1	B2M, CIITA, TRAC-Knock Out	Acute Lymphoblastic Leukemia, Chronic Lymphocytic Leukemia, Non Hodgkin Lymphoma	CD19-specifc CAR-T cells
NCT04037566	1	HPK1-Knock Out	Acute Lymphoblastic Leukemia in Relapse Acute Lymphoblastic Leukemia Refractory Lymphoma, B-Cell CD19-Positive	XYF19 CAR-T cells
NCT04557436	1	CD52 and TRAC-Knock Out	B-Cell Acute Lymphoblastic Leukemia	CD19-specifc CAR-T cells
NCT04976218	1	TGFβR-Knock Out	Advanced Biliary Tract Cancer	CAR-EGFR T cells
NCT04767308	1 (early)	CD5-Knock Out	CD5+ Relapsed/ Refractory Hematopoietic Malignancies Chronic Lymphocytic Leukemia, Mantle Cell Lymphoma, Diffuse Large B-cell Lymphoma, Follicular Lymphoma, Peripheral T-cell Lymphomas	CT125A cells
NCT04244656	1	Uncharacterized	Multiple Myeloma	Anti-BCMA Allogeneic CRISPR-Cas9Engineered T Cells (CTX120)
NCT04438083	1	Uncharacterized	Renal Cell Carcinoma	Allogeneic CRISPRCas9-Engineered T Cells (CTX130)
NCT04502446	1	Uncharacterized	T-Cell Lymphoma	Allogeneic CRISPRCas9 Engineered T Cells (CTX130)
NCT04035434	1	Uncharacterized	B-cell Malignancy Non-Hodgkin Lymphoma B-cell Lymphoma Adult B-Cell Acute Lymphoblastic Leukemia	CD19-specifc CAR-T cells (CTX110)
NCT04637763	1	Uncharacterized	Lymphoma Non-Hodgkin Lymphoma Relapsed Non Hodgkin Lymphoma Refractory B-Cell Non Hodgkin Lymphoma	CD19-specifc CAR-T cells (CB-010)
NCT03398967	1/2	Uncharacterized	B-Cell Leukemia, B-Cell Lymphoma	Universal Dual Specificity CD19 and CD20 or CD22 CAR-T Cells
NCT05066165	1/2	Uncharacterized	Acute Myeloid Leukemia	WT1-directed TCR T cells
NCT04244656	1	Uncharacterized	Multiple Myeloma	Anti-BCMA Allogeneic CRISPR-Cas9Engineered T Cells (CTX120)

## Advancements in CRISPR-Cas9 technology

In recent years, the area of genome engineering has seen tremendous improvements in gene-editing technology. Base editing and prime editing are two such innovations that enables exact DNA sequence alterations without the requirement for double-stranded breaks or reliance on cellular repair processes.

### Base editing

Base editing is a versatile genome editing method introduced by David Liu and his team in 2016, which enables for the direct modification of individual nucleotides in the DNA sequence without requiring the introduction of DSBs or HDR mechanisms^30,31,92,93^. It is based on the fusion of a catalytically inactive CRISPR-associated protein (dCas9) with a cytidine or adenine deaminase enzyme. These enzymes facilitate the conversion of cytosine (C) to uracil (U) or adenine (A) to inosine (I), respectively, within a specific target site. Base editors are a type of genetic editing tool that involves the use of a dCas9 protein, a cytidine or adenine deaminase enzyme, and a guide RNA molecule. The cytidine deaminase enzyme, typically derived from APOBEC (apolipoprotein B mRNA editing enzyme, catalytic polypeptide-like) proteins or AID (Activation-Induced Cytidine Deaminase), converts cytidine (C) to uridine (U) within a specific editing window. There are various base editing techniques, including cytosine base editors (CBEs) and adenine base editors (ABEs), each with its mechanism^30,31,94^.

**Figure 5:**
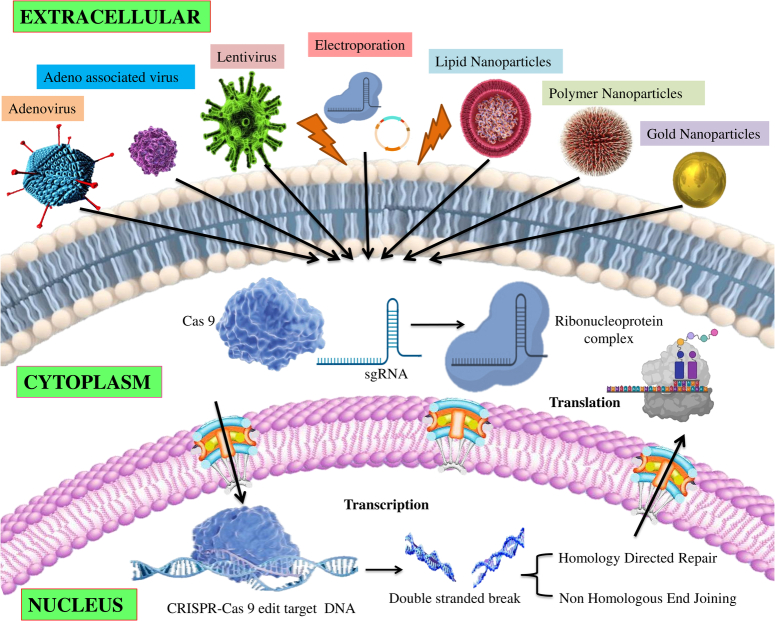
Mechanisms of (A) Base Editing and (B) Prime Editing.

CBEs uses a cytidine deaminase enzyme, uracil DNA glycosylase to convert a specific DNA base, cytosine (C), to another base, uracil (U), while the DNA is still in its single-stranded state. The CBE system consists of a Cas9 protein, a single guide RNA (sgRNA), and a DNA repair template. The sgRNA guides Cas9 to the target site, where it unwinds the DNA double helix. The cytidine deaminase domain deaminates cytosine to uracil, which is converted to thymine during DNA replication or repair, resulting in a permanent C-to-T conversion in the genome (Fig. 5). This method can be employed to rectify genetic diseases like sickle cell anemia and cystic fibrosis, which result from individual genetic mutations, without harming other sections of the genetic material.

ABEs use an adenine deaminase enzyme to convert a specific DNA base, adenine (A), to another base, inosine (I), while the DNA is still in its single-stranded state. The inosine is recognized as a guanine (G) base by the cell’s natural DNA repair mechanisms, resulting in an A to G conversion. To achieve adenine to guanine conversion, ABEs consist of three main components: a Cas9 variant (dCas9), which lacks nuclease activity and serves as a DNA targeting platform, a cytidine deaminase (APOBEC enzymes), which catalyzes the conversion of adenine to inosine (I), and a DNA repair inhibitor such as uracil DNA glycosylase inhibitor (UGI), which prevents the cellular repair machinery from reverting the inosine to adenine. The inosine is recognized as guanine during DNA replication, resulting in a permanent A-to-G conversion (Fig. IV)^30,31,94^. This approach can be used to correct genetic mutations that cause diseases such as hereditary hearing loss.

## Benefits and limitations of base editing

Base editing creates precise single-nucleotide changes, avoiding DSBs, improving product purity and reducing indels. It is found that base editing may correct over 70% of SNPs which are disease-related^95^. Base editing offers potential for treating single-point mutation-related genetic diseases like sickle cell anemia and cystic fibrosis, while also aiding researchers in understanding gene function by precisely altering nucleotides in model animals. Base editing is a promising approach, but it has limitations such as its ability to change one DNA base at a time, making it unsuitable for all genetic mutations, and its targeting effectiveness may change based on the DNA sequence, reducing precision and specificity^96–99^. Ongoing research is focused on improving the efficiency, specificity, and safety of base editing techniques to make them more effective in clinical applications.

### Prime editing

Prime editing is a recent gene editing method that allows precise changes to the genome without damaging the DNA molecule’s two strands. It uses reverse transcriptase enzymes and proteins to target DNA sequences, inserting new DNA sequences at the target site using an RNA template. Prime editing can cure a wider range of genetic mutations without damaging other regions of the genome^93^. Prime editing, introduced by Liu and his team in 2019, is a more accurate and less likely method for modifying genetic bases in human cells without the need for DSBs or donor DNA templates, offering a more efficient and accurate approach^65^. It involves a prime editing guide RNA (pegRNA), an engineered Cas9 nickase (H840A), and a reverse transcriptase (RT). The pegRNA consists of three regions: a scaffold, a primer binding site (PBS), and an editing template (ET). The pegRNA binds to the target DNA strand using complementary base pairing and the scaffold region. The RT creates a complementary DNA sequence by hybridizing the PBS region with the target DNA. The required edit is present in the pegRNA’s ET region and reverse transcribed using RT-mediated synthesis. As a consequence, a chimeric DNA molecule is produced, and Cas9 nickase nicks the nonedited DNA strand to complete the primary editing process (Fig. 5)^31,65,100^. Prime editing offers a more flexible and precise alternative to conventional CRISPR-Cas9 techniques, as it allows for precise alterations like point mutations, insertions, deletions, and extensive edits, despite the limited off-target consequences.

## Benefits and limitations of prime editing

Prime editing is a novel method of genome editing that directly modifies the DNA sequence, improving accuracy and reducing the risk of unintended mutations or off-target effects. It can correct point mutations, small insertions, and deletions, common causes of genetic diseases, and can edit multiple sites simultaneously, enabling complex genetic disorders correction. Prime editing is a safer alternative to traditional CRISPR-Cas9 methods, as it minimizes the reliance on DNA repair mechanisms^101^. It has lot of promise but has certain limitations, including difficulty in distributing primary editing components into target cells, particularly *in-vivo*. Researchers are developing delivery techniques like viral vectors or LNPs to improve their effectiveness and safety in various cell types and tissues. The efficiency of prime editing is variable and depends on the precise genomic region being edited. To increase efficiency and accuracy, researchers are exploring Cas9 variations and optimizing pegRNA design^65,101,102^.

## Challenges and limitation of CRISPR-Cas9

CRISPR-Cas9, despite its significant advancements, still faces numerous challenges and limitations that must be addressed before its clinical application can be considered. These challenges must be addressed safely and effectively to fully utilize CRISPR-Cas9’s potential, making it crucial to recognize and overcome these obstacles.

### Off-Target effects

CRISPR-Cas9, a tool designed to target specific genes, can unintentionally modify other genes, leading to off-target effects and posing a significant concern in *in-vivo* therapeutic applications^103,104^. The off-target effects of CRISPR-Cas9 are influenced by factors like guide RNA length, target DNA sequence, and Cas9 enzyme efficiency. Studies show off-target effects can occur with highly specific guide RNAs, and different Cas9 enzymes have varying off-target profiles^105^. Researchers are striving to create methods to reduce off-target effects, which are a major worry for the safety and effectiveness of CRISPR-Cas9. Various *in silico* detection have successfully detected and predicted off-target cleavages, but their ability to anticipate epigenetic alterations is limited to homologous genes. Technological advancements like high-throughput genome-wide next-generation sequencing are crucial for reducing off-target effects^104,106^. CRISPR-Cas9 has been reduced off-target using high-fidelity Cas9 enzymes and alternative gene-editing technologies like base editors or prime editors, Cas9 nickases, which have been shown to have fewer off-target effects^107,108^. The off-target effects of CRISPR-Cas9 remain a significant concern, prompting researchers to conduct further research to understand their mechanisms and develop new methods for their reduction, which is crucial for ensuring the safety and effectiveness of CRISPR-Cas9 in future applications.

### Delivery efficiency and targeting

The successful gene editing process requires efficient transport of CRISPR-Cas9 components to target cells and tissues. The delivery method should be effective, minimally immunogenic, and capable of transporting Cas9/sgRNA to specific cells or organs. Various methods, such as viral vectors, electroporation, and lipofection, have limitations and can be inefficient or toxic to cells. However, plasmid-based expression has been effective for *in-vivo* application using hydrodynamic injection or electroporation^14,109–111^ but these target delivery are limited and have poor Cas9 activity control. Therefore, viral and nonviral delivery methods, such as AAV, have been developed for gene therapy due to their compatibility with most human populations and high transduction efficiency^112–115^ and can be apply directly to the target organ or can apply systemically^115–118^. But the limitation in AAVs delivery system is due to their limited cargo size^113^. Clinical trials use viral vectors like adenovirus and lentivirus, but lentiviral vectors have safety issues and immunogenicity, making them unsuitable for therapeutic use due to their drawbacks^119,120^. Another approach for delivery system is the no-viral method that used LNPs, which are low cost with high compatible^114,121^. However, nanoparticles carriers have low editing efficiency^122^. Numerous studies have investigated the efficiency and specificity of CRISPR-Cas9, with one utilizing a modified version of the Cas9 protein to precisely target specific genes, minimizing off-target effects^123^. Another study used nanoparticles as a delivery method, which showed improved delivery efficiency and reduced toxicity compared to other methods^124^. A recent study used LNPs for delivery of mRNAs and siRNAs with antibody targeted cell specific delivery^125–127^. A study found that injecting CRISPR LNPs targeting PLK1 (referred to as sgPLK1-cLNPs) in metastatic orthotopic glioblastoma led to an ~70% improvement in *in vivo* gene editing accuracy, a 50% reduction in tumor growth, cell death, and a 30% extension in survival^128^. The study also found that intraperitoneal injections of sgPLK1-cLNPs, targeting EGFR, significantly improved the precision of in vivo gene editing for scattered ovarian cancers by 80%. Therefore, nonviral delivery methods like microinjection, transfection, and electroporation are promising carriers for gene editing. Electroporation creates nano-sized pores in cell membranes using high-voltage currents. Studies have shown successful delivery of Cas9-gRNA complexes in mammalian cells using electroporation or transfection using liposomes^129,130^. Further they found that the life half of the Cas9-gRNA ribonucleoprotein complex was longer in lentiviral transduction or plasmid DNA transfection as compared to electroporation delivery^129^.

### Large DNA fragment insertion and deletion

The Cas9 enzyme’s ability to insert or delete DNA fragments is limited by its size, which can cut DNA at specific sites. The CRISPR-Cas9 technology often struggles to insert or delete segments larger than 1–2 kb, and the efficiency of the procedure remains a significant limitation. The CRISPR-Cas9 system is effective for small edits like point mutations or insertions/deletions, but less effective for larger modifications due to the difficulty for the Cas9 enzyme to locate and cut the correct location in the DNA^25^. The Cas9 enzyme can cause off-target effects when working with larger DNA fragments, potentially altering the genome without user consent. As it becomes more challenging to verify the enzyme’s precise cutting location, this risk increases as larger DNA fragments are used^68^. The CRISPR-Cas9 system has limitations in large DNA fragment insertion and deletion, but potential solutions include using other genome editing tools like HR or transposons, or altering the Cas9 enzyme to increase its ability to precisely cut larger DNA pieces^131^.

### Mosaicism and heterogeneity

When CRISPR-Cas9 is used for gene editing, various cells within an organism or tissue may show varied degrees of genetic change, a condition known as mosaicism. Mosaicism refers to the presence of more than one genotype in an organism. Mosaicism can develop naturally or can be produced using a variety of methods, such as CRISPR-Cas9^132^. Mosaicism can lead to undesired mutations in the genome, potentially compromising the precision of CRISPR-Cas9 gene editing, potentially leading to unanticipated consequences like illness emergence, making it challenging to produce regular and predictable results due to this variation in editing efficiency. Variations in the CRISPR-Cas9 system’s delivery, timing, or effectiveness may result in mosaicism^132^. According to studies, CRISPR-Cas9 can cause mice to develop tumors and experience other negative consequences by inducing mosaicism in their genomes^109^. The study revealed that CRISPR-Cas9 can cause off-target DNA alteration, highlighting the need to understand and reduce mosaicism to develop reliable and repeatable gene-editing methods. On the other side, heterogeneity describes the variance in an organism’s genome. Heterogeneity can affect the accuracy of CRISPR-Cas9 gene editing by introducing undesirable mutations and making it difficult to achieve consistent results due to the potential changes in an organism’s genome due to factors like age and environmental conditions. A study revealed that an organism’s genome’s heterogeneity can impact the accuracy of CRISPR-Cas9 gene editing, potentially introducing unwanted mutations and causing unpredictable effects on the organism, in the research^133^. Researchers are exploring ways to overcome the limitations of CRISPR-Cas9, including using other gene editing techniques like base and prime editing, and combining CRISPR-Cas9 with methods like gene therapy and medication delivery to mitigate the negative effects of mosaicism and heterogeneity.

### Ethical and regulatory considerations

The ethical issue of CRISPR-Cas9 is its potential for unexpected repercussions due to its infancy and lack of understanding of its operation. The potential long-term implications of genetic modification, such as altering a creature’s genetic makeup, could be unforeseen, potentially impacting other living things in the ecosystem and potentially causing negative mutations on the transformed creature^134^. CRISPR-Cas9 technology raises ethical concerns about potential misuse. It could be used to create genetically modified organisms for military purposes or biofuels, but also for more nefarious purposes like creating biological weapons or designing organisms to spread disease^135^. Regulatory considerations are crucial in the use of CRISPR-Cas9. There are debates about the appropriate use and purpose of this technology, with some advocating for its exclusive use by highly trained professionals, while others advocate for its widespread availability to researchers and amateur scientists. Additionally, there are concerns about ensuring safe and responsible use of CRISPR-Cas9^135^. The ethical and regulatory limitations of CRISPR-Cas9 are crucial when using this technology. Despite its potential benefits, it is crucial to weigh the risks against the benefits to ensure responsible and safe use of this powerful tool.

### Immune response and immunogenicity

CRISPR-Cas9’s immunogenicity is a concern as it can trigger an immune reaction, potentially reducing gene editing efficiency and causing harm when reintroduced into the bloodstream. Strategies to reduce immunogenicity include altering the Cas9 protein, which can decrease immunogenicity while increasing efficacy, and using immuno-suppressive medications to prevent the immune system from recognizing and attacking CRISPR-Cas9. Addressing this issue is crucial for effective gene editing^91^. A study showed that CRISPR-Cas9 can elicit an immune response in mice, leading to the production of antibodies against Cas9^136^. Various strategies have been proposed to reduce the immunogenicity and immune response restrictions of CRISPR-Cas9. One approach involves altering the Cas9 protein, which can decrease immunogenicity while increasing efficacy. Another approach involves using immuno-suppressive medications to prevent the immune system from recognizing and attacking CRISPR-Cas9^137^. The patient’s immune system may be weakened by this method, which might have negative implications. Several studies show an immune response against the Cas9 protein and antibody development against CRISPR-Cas9. Strategies like protein modification and immune-suppressive drugs are proposed to overcome these limitations.

### Application in Nondividing cells

The CRISPR-Cas9 system faces challenges in nondividing cells due to limited delivery methods. Neurons and muscle cells, which are examples of nondividing cells, have low DNA uptake rates, making it difficult to introduce foreign DNA, and limiting its application in certain tissues and organs. Therefore, effective delivery methods are crucial for successful application^138^. The CRISPR-Cas9 system faces a limitation in nondividing cells due to its low efficiency in gene editing, as it relies on the cell’s own repair mechanisms for DNA breaks, which are less active in these cells, limiting the system’s precision and accuracy. Furthermore, the CRISPR-Cas9 system, particularly in nondividing cells, can cause off-target effects due to extended editing DNA persistence, increasing the risk of unintended consequences and potentially limiting the system’s safety and efficacy. Researchers have developed strategies to improve the application of the CRISPR-Cas9 system in nondividing cells, including using viral vectors like AAVs. AAVs efficiently transduce a range of nondividing cells, including neurons and muscle cells, making them an attractive delivery method^139^. Another strategy is to use alternative nucleases like base editors and prime editors that are more efficient and precise than the traditional CRISPR-Cas9 system for gene editing in nondividing cells, as they do not require DNA breaks for gene editing^65^.

## Conclusion

CRISPR-Cas9 technology has revolutionized cancer research by providing a powerful tool for precise gene editing. It has shown promise in improving cancer treatment outcomes, identifying cancer-related genes, verifying medicinal targets, and creating complex cancer models for understanding carcinogenesis. This technique allows precision oncology and personalized medicine by enabling the creation of tailored treatments based on a person’s genetic profile. It can also fix disease-causing mutations and enhance existing treatments, such as immunotherapies. By altering immune cells’ genes, researchers can enhance tumor recognition and survival, potentially increasing the effectiveness of immunotherapies and broadening their use for various types of cancer. CRISPR-Cas9 gene editing has made significant progress, but challenges like off-target impacts and delivery strategies need to be addressed for safety and specificity. Current research aims to improve precision, efficiency, and ethical considerations. Discussions around germline editing and potential unintended consequences are crucial. The future of CRISPR-Cas9 research in cancer holds hope for improved accuracy, combination medicines, and interdisciplinary partnerships. However, utilizing CRISPR-Cas9’s transformative potential requires ongoing research, collaboration, and careful ethical consideration. Conducting this will lead to a less prevalent cancer, tailored treatments, and a significant reduction in the burden of cancer. This review intends to inspire more research and partnerships in utilizing the full potential of CRISPR-Cas9 for enhancing cancer understanding and creating innovative treatment approaches by emphasizing the present advancements, challenges, and future possibilities.

## Ethical approval

N/A. Ethical approval was not necessary since it is a review manuscript and does not involved using original human or animal.

## Consent

N/A. Consent form was not required since it is a review manuscript.

## Sources of funding

Nil.

## Author contribution

S.N.D.: writing – original draft and writing – review and editing; R.R.: conceptualization, investigation, supervision, validation, and writing – review and editing; P.M.: visualization; N.K.G.: visualization and writing – review and editing.

## Conflicts of interest disclosure

The authors declare no conflicts of interest.

## Research registration unique identifying number (UIN)

1. Name of the registry: not applicable.
2. Unique identifying number or registration ID: not applicable.
3. Hyperlink to your specific registration (must be publicly accessible and will be checked): not applicable.

## Guarantor

Shamjetsabam Nandibala Devi, Rashmi Rana, Priyanka, and Nirmal Kumar Ganguly.

## Data availability statement

N/A. No report results derived from research data was used.

## Provenance and peer review

Invited.
